# Comparative insights into the metabolite and taste discrepancies upon kimchi (*Mukeunji*) fermentation using different *Lactobacillaceae* starters

**DOI:** 10.1016/j.fochx.2025.102982

**Published:** 2025-09-02

**Authors:** Moeun Lee, Daun Kim, Jung Hee Song, In Min Hwang, So Yeong Mun, Ji Yoon Chang

**Affiliations:** a Fermentation Systems Research Group, World Institute of Kimchi, Gwangju 61755, Republic of Korea; b Department of Food and Nutrition, Chosun University, Gwangju 61452, Republic of Korea; c Division of Applied Life Science (BK21), Institute of Agriculture and Life Science, Gyeongsang National University, Jinju 52828, Republic of Korea; d Institute of Smart Space Agriculture (ISSA), Gyeongsang National University, Jinju 52828, Republic of Korea

**Keywords:** Kimchi starter, Metabolite profile, Electronic tongue, Electronic nose, Sensory evaluation, *Companilactobacillus allii* WiKim39, *Lactococcus lactis* WiKim0124, *Latilactobacillus sakei* WiKim0176

## Abstract

Starter cultures play a key role in shaping the flavor and sensory traits of kimchi during fermentation. This study compared seven *Lactobacillaceae* starters using metabolite profiling and electronic tongue/nose analyses. A total of 46 key compounds were identified via UHPLC-Q-Orbitrap-MS and HPLC. Principal component analysis showed that metabolite and sensory changes were mainly influenced by specific starters and fermentation stages rather than species or genus. Amino acid derivatives, including glutamic acid, aspartic acid, γ-glutamic acid, serine, and γ-aminobutyric acid, showed differing concentrations depending on the starter and were associated with umami perception. Electronic tongue results indicated increased sourness and decreased sweetness and bitterness across all samples, while umami notably increased only with a specific starter. Aroma compounds detected by the electronic nose also followed starter- and time-based patterns. These findings provide practical guidance for selecting effective starter cultures to enhance the umami-rich flavor of aged kimchi (*Mukeunji*).

## Highlights

• Seven Lactobacillaceae starters were evaluated for their impact on kimchi fermentation.
• Metabolite profiling identified 49 key compounds related to taste and aroma changes.
• Umami-related compounds varied depending on the specific starter strain used.
• Starter strains reduced off-flavors and enhanced sour aroma under acidic conditions.

## 1 Introduction

The overall quality of kimchi is primarily determined by multiple factors that impact its physical, chemical, and microbiological characteristics, all of which are critical in evaluating its quality (Lee, Kim, Hwang, et al., 2024). Taste is one of the most essential factors as it is vital in determining food quality. Kimchi is produced by the natural fermentation of vegetables combined with various spices and ingredients, and the bacterial community plays a significant role in enhancing the complexity of its flavor and aroma (Portaro et al., 2022). Specific starters are introduced to optimize this process, regulate the microbial community during fermentation, and improve the overall quality and sensory attributes of kimchi (Lee et al., 2020).

In industrial kimchi production, starter cultures are crucial role determining the quality by introducing different lactic acid bacteria (LAB) strains, each capable of metabolizing various substrates to produce diverse flavor compounds (Jung et al., 2025). Many volatile flavor compounds are derived from the breakdown of proteins, carbohydrates, and amino acids and their production is significantly higher when starter cultures (Jung et al., 2022). Consequently, the use of specific LAB starters for kimchi production improves the sensory characteristics and functional properties. This approach is widely recognized as an effective solution to overcome the challenges of kimchi production.

Numerous studies have reported that different starters produce distinct tastes. It is widely recognized that using heterofermentative strains such as *Leuconostoc mesenteroides* isolates as starters leads to the rapid production of mannitol, a natural sugar alcohol found in fruits and vegetables that is mildly sweet and contributes to a refreshing flavor (Moon et al., 2025). However, owing to the specific properties of certain L. *mesenteroides* starters, such as variations in malic acid levels, a more targeted selection of starter strains can influence the development of diverse flavor profiles in kimchi (Kang et al., 2024). In addition, LAB succession typically occurs during fermentation, with *Leuconostoc* species dominating the early and middle stages and *Lactobacillus* species becoming predominant in the later stages (Lee et al., 2017).

*Lactobacillaceae* starters are vital in shaping the taste profiles of various fermented foods through their metabolic activities. These bacteria contribute to the production of lactic acid, which lowers the pH and imparts a tangy or sour flavor commonly observed in foods such as yogurt (Celik et al., 2021), sauerkraut (Zhou et al., 2023), and kimchi (Ko et al., 2024). As pH decreases during fermentation, the acid resistance of the starter strain becomes a critical factor for its survival in kimchi. This tolerance contributes to the development of the distinctive flavor and texture of *Mukeunji* (long-term fermented kimchi), distinguishing it from overripe kimchi, which is often associated with an overly sour taste, soft texture, and off-flavors (Mun et al., 2024). However, the metabolic traits of *Lactobacillaceae* vary significantly depending on the strain. While some strains contribute positively to the flavor and acid balance in kimchi, others may overproduce organic acids or develop off-flavors, which can adversely affect the optimum ripening period and sensory quality (Lee, Kim, et al., 2024). Consequently, their application in the kimchi industry is limited, and further research is required to identify suitable strains with desirable fermentation characteristics.

The objective of the present study was to compare the flavor, aroma perception, and metabolite composition of fermented kimchi produced using seven isolates of *Lactobacillaceae* starters with different fermentation characteristics and a reference starter strain, WiKim0124, which was shown to exhibit superior flavor and sensory characteristics in our previous study (Lee, Kim, Hwang, et al., 2024). Liquid chromatography-tandem mass spectrometry (LC-MS/MS) was employed to investigate taste-related compounds produced by starter cultures during kimchi fermentation. Comprehensive metabolomic profiling revealed distinct patterns of organic acids, sugars, and amino acids associated with each starter strain. Sensory characteristics were further evaluated using electronic tongue (E-tongue) and electronic nose (E-nose) technologies. These integrated findings highlight how different *Lactobacillaceae* starter strains shape the flavor, aroma, and metabolite landscape of kimchi, offering valuable insights for selecting optimal strains to produce premium-quality *Mukeunji*.

## 2 Materials and methods

### 2.1 Bacterial strains

Seven *Lactobacillaceae* strains, including *Lactiplantibacillus plantarum* LP8, *Lactiplantibacillus plantarum* WiKim0189, *Latilactobacillus sakei* WiKim0190, *Latilactobacillus sakei* WiKim0176, *Latilactobacillus curvatus* LC102, *Latilactobacillus curvatus* LCM2, and *Companilactobacillus allii* WiKim39 (GenBank ID: NR_159087.1), were isolated from kimchi and used in this study. Additionally, *Lactococcus lactis* WiKim0124 (GenBank ID: MZ424472.1) previously isolated from kimchi (Lee et al., 2023) was used as a reference strain (Table 1).

**Table 1:** Experimental kimchi samples.

Strain	Gene bank ac no.	Code
*Lactiplantibacillus plantarum* LP8	–	LP8
*Lactiplantibacillus plantarum* WiKim0189	–	WiKim0189
*Latilactobacillus sakei* WiKim0190	–	WiKim0190
*Latilactobacillus sakei* WiKim0176	–	WiKim0176
*Latilactobacillus curvatus* LC102	–	LC102
*Latilactobacillus curvatus* LCM2	–	LCM2
*Companilactobacillus allii* WiKim39	NR_159087.1	WiKim39
*Lactococcus lactis* WiKim0124	MZ424472.1	WiKim0124

### 2.2 Preparation of starter kimchi samples

Kimchi cabbage and minor ingredients, including red pepper powder, ginger, and garlic, used for kimchi preparation were sourced from a local grocery store. Salted Kimchi cabbage was mixed with these vegetables, and kimchi was prepared according to the ratios described in a previous study. Detailed percentage ratios of the ingredients used in the kimchi recipe are provided in Table S1 of a previous study (Lee, Kim, Hwang, et al., 2024). Kimchi samples were categorized into eight groups: One control group without starter addition (CTL) and seven groups inoculated with different *Lactobacillaceae* starter strains—LP8, WiKim0189, WiKim0190, WiKim0176, LC102, LCM2, and WiKim39—were used, with *Lactococcus lactis* WiKim0124 included as a reference strain. The starters were incorporated into the kimchi sauce at a concentration of 10^7^ colony-forming units (CFU) per gram of kimchi weight. Each sample, with or without starter cultures, was packed into 1-kg polyethylene plastic bags. The samples were stored at 6.0 ± 0.5 °C for four weeks, with microbial and chemical changes monitored regularly to track the fermentation progression.

### 2.3 Chemical analyses

Each kimchi sample, weighing 500 g, was homogenized using a hand blender (model HR1372/90; Philips, Andover, MA, USA) and filtered through a sterilized gauze. The pH of the resulting filtrate was measured using a pH meter (Orion 3-Star; Thermo Scientific, Waltham, MA, USA). Next, the kimchi filtrate was titrated with 0.1 N NaOH until the pH reached 8.3 to determine the titratable total acidity (%). Total acidity was calculated using the following formula: Total acidity (% lactic acid) = (Volume of 0.1 N NaOH [mL]) × (Equivalence factor of 0.1 N NaOH solution) × (Organic acid constant corresponding to 1 mL of 0.1 N NaOH [where lactic acid = 0.009]) × 10.

### 2.4 Enumeration of the total viable cell and LAB counts in kimchi samples

Kimchi filtrate was serially diluted using a 0.85 % (*w*/*v*) physiological saline solution. The diluted samples were inoculated onto the plate count agar (Difco, Detroit, MI, USA) for total viable bacteria enumeration and de Man, Rogosa, and Sharpe agar (Difco) supplemented with 2 % (w/v) CaCO₃ for LAB enumeration. The plates were incubated at 30 °C for 48 h, and microbial counts were recorded as CFU per mL.

#### 2.4.1 Analysis of starter dominance

Bacterial community analysis of kimchi starter strain abundance was performed following a previously established method (Jeong et al., 2024). Total DNA was extracted using the DNeasy PowerSoil Kit (Qiagen) and quantified using Quant-IT PicoGreen (Invitrogen). The Illumina 16S Metagenomic Sequencing Library was prepared to amplify the V3 and V4 regions, followed by polymerase chain reaction amplification with Herculase II fusion DNA polymerase (Agilent) and NexteraXT Indexed Primers for final library construction. The library was quantified via quantitative polymerase chain reaction (KAPA Library Quantification Kit) and qualified using the TapeStation D1000 ScreenTape (Agilent). Paired-end sequencing (2 × 300 bp) was performed using the MiSeq platform (Illumina).

### 2.5 Targeted metabolomics analysis

#### 2.5.1 Ultra-high-performance liquid chromatography (UHPLC)-heated electrospray ionization-quadrupole (Q)-Orbitrap tandem mass spectrometry (MS/MS)

Fourteen organic acids, one sugar alcohol, two sugars, and 17 amino acids—previously identified as key metabolites associated with LAB-driven kimchi fermentation (Lee, Kim, Hwang, et al., 2024)—were simultaneously analyzed using UHPLC-Q-Orbitrap-MS. The analysis was conducted using a validated processing method described in a previous study (Jeong et al., 2023). All reference standards used in this study were sourced from Sigma-Aldrich Co. (St. Louis, MO, USA). Detailed analytical conditions and fragmentation patterns are presented in Tables S1–2.

#### 2.5.2 HPLC

Concentrations of organic acids and sugars in kimchi were monitored throughout the fermentation period using the Waters Alliance e2695 system (USA) equipped with the Sugar Pak (WAT085188) column. The column oven temperature was maintained at 95 °C, with distilled water as the mobile phase. The total run time was 25 min, with a flow rate of 0.5 mL/min. Detection was performed using a refractive index detector, and injection volume was set to 20 μL to ensure accurate quantification.

### 2.6 Evaluation of sensory quality

#### 2.6.1 E-tongue

Kimchi filtrates, diluted 20-fold, were used as samples for taste assessment through E-tongue analysis using the α-Astree II electronic tongue system (Alpha MOS, Toulouse, France), featuring a sensor array composed of seven chemical sensors: AHS (sourness), NMS (umami), CTS (saltiness), ANS (sweetness), SCS (bitterness), and PKS and CPS (comprehensive taste), along with an Ag/AgCl reference electrode. Each analysis was conducted in triplicate to ensure accuracy and reproducibility.

#### 2.6.2 Electronic nose

Undiluted kimchi filtrates served as samples for aroma evaluation using electronic nose analysis. Volatile compounds in the filtrates were assessed using a fast gas chromatography electronic nose system (Heracles II model; Alpha MOS), following a slightly modified analytical procedure (Lee, Kim, et al., 2024). All procedures were repeated three times for consistency.

### 2.7 Statistical analysis

Principal component analysis (PCA) was conducted using the “prcomp” function from the “ggfortify” package in R (v3.3.2; https://www.r-project.org/). Group comparisons were performed using the Tukey's honest significant difference test with the “agricolae” package, whereas non-parametric (Spearman's) correlations were assessed using the “corrplot” function from the “PerformanceAnalytics” package. A significance threshold of *p* < *0.05* was used for statistical analysis. Based on the PCA results, kimchi samples were categorized into three distinct groups (Group I, II, and III) according to their metabolite profiles and fermentation progression.

## 3 Results and discussion

### 3.1 Physicochemical and microbial properties with starter domination ratio

**Fig. 1:**
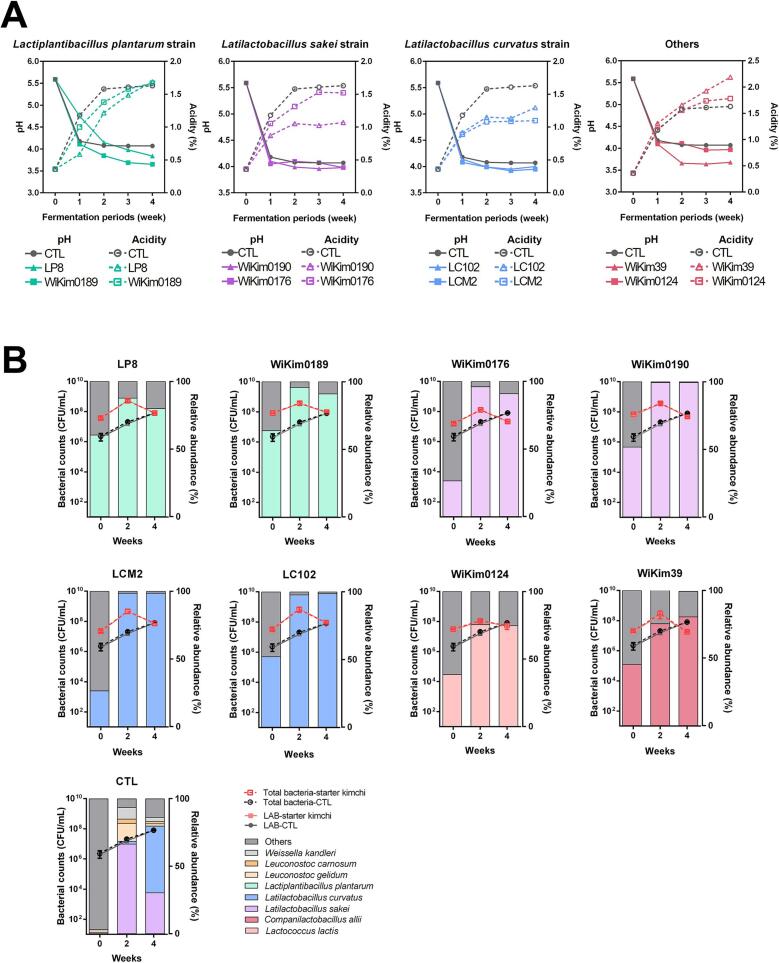
Characteristics of starter kimchi throughout fermentation. pH profile, titratable acidity (A), total bacterial count, lactic acid bacteria (LAB) count, and analysis of the starter proportions in kimchi (B). The starter dominance ratio was calculated as the ratio of inoculated starters to total viable bacteria (B). All experiments were conducted in triplicate.

LAB starters are essential in kimchi fermentation, as they suppress undesirable microbes through pH reduction and metabolite production. An optimal starter that drives fermentation enhances stability and improves the final product's quality and sensory acceptance (Shao et al., 2024). As shown in Fig. 1A, the initial pH of all the kimchi samples was 5.59, which decreased rapidly during fermentation. The pH values ranged from 4.05 to 4.75 on day 7, and declined further to 3.65 to 3.98 on day 28, indicating active fermentation. Most starter kimchi samples maintained a pH between 3.90 and 4.00. WiKim0189 and WiKim39 had the lowest pH values of 3.65 and 3.68, respectively. Total acidity rose from 0.36 to 0.56–1.31 % on day 7 and 1.05–1.66 % on day 14, confirming organic acid production. The inoculated samples showed higher acidity than the control, highlighting the strain-specific effects of *Lactobacillaceae* starters on acidification. In the non-starter kimchi (CTL), *L. sakei* was initially dominant on day 14, but *L. curvatus* later became the predominant strain. The total bacterial count at the start of fermentation was 2.3 × 10^6^ CFU/mL, whereas the LAB count was 1.9 × 10^6^ CFU/mL. The total viable bacterial count significantly increased during fermentation. On day 0, bacterial count in the inoculated group ranged from 2.2 × 10^7^ to 8.3 × 10^7^ CFU/mL, rising to 2.3 × 10^8^ to 3.3 × 10^8^ CFU/mL on day 7, indicating microbial growth and adaptation (Fig. 1B). The LAB count followed a similar trend, increasing from 3.3 × 10^7^ to 2.3 × 10^8^ CFU/mL on day 7, with the highest count observed for WiKim0190. The microbial composition and fermentation characteristics of kimchi are initially influenced by starter inoculation. However, as fermentation progresses and starter dominance declines, the microbial community gradually resembled that of non-inoculated kimchi, making it challenging to identify the specific effects of inoculation (Park et al., 2019). Starter strains with occupancy rates exceeding 70 % during the late fermentation stage were selected in this study to accurately assess their impact on the kimchi fermentation dynamics and address this. LAB populations remained stable or slightly increased on day 14, reaching 75–99 % and demonstrating sustained bacterial activity. Starter population ratios varied among the strains, with L. *curvatus* starters showing the highest dominance at 98.6 % on day 28, followed by L. *sakei* starters at 91.0–99.4 %. The inoculated samples sustained a starter ratio above 70 % throughout fermentation, demonstrating strong colonization and fermentation potential. Therefore, these strains are regarded as suitable fermentative microorganisms with the capacity to enhance product quality through metabolite production during kimchi fermentation.

### 3.2 Sensory perception

#### 3.2.1 Metabolic trends in starter kimchi

**Fig. 2:**
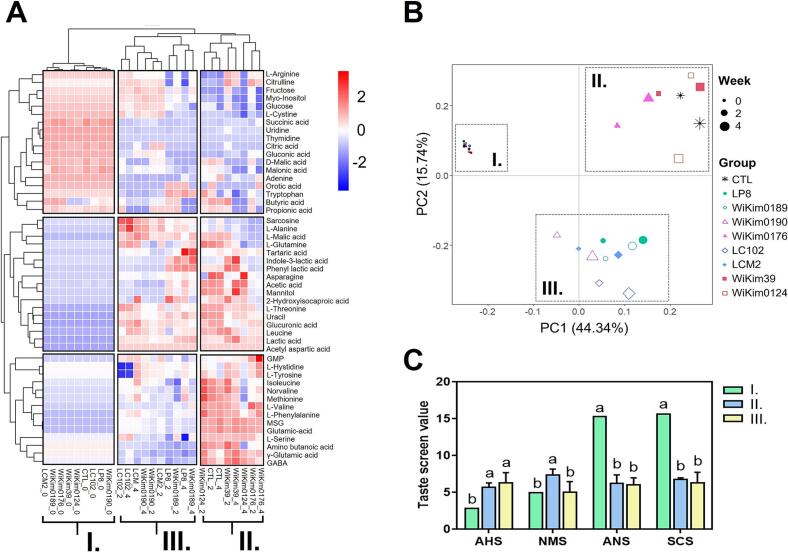
Comparison of the metabolite compositions and sensory properties of different Kimchi samples. Hierarchical clustering of 46 kimchi metabolites identified via high-performance liquid chromatography (HPLC) and LC-quadrupole (Q)-Orbitrap mass spectrometry (MS) is shown in the heatmap. The colour scheme illustrates the compound abundance, with red indicating elevated compound levels and blue indicating reduced compound levels (A). Principal component analysis (PCA) plot (B). Sensory attribute profiles: SCS, bitterness; CTS, saltiness; NMS, umami; ANS, sweetness; AHS, sourness (C). Samples were classified into three groups: Group I, 0-week samples; Group II, a subset of 2- and 4-week samples (WiKim0176, WiKim39, WiKim0124, and control); and Group III, another subset of 2- and 4-week samples (LP8, WiKim0189, WiKim0190, LC102, and LCM2). Different letters denote the significant differences (*p* < 0.05; analysis of variance [ANOVA], followed by the Tukey honest significant difference [HSD] test). All experiments were conducted in triplicate. (For interpretation of the references to colour in this figure legend, the reader is referred to the web version of this article.)

The complex and rich flavor of kimchi results from the interactions and combinations of its metabolites, making investigation of starter-derived metabolites crucial for understanding the distinctive flavor profiles each starter contributes to the fermentation process. A total of 46 metabolites, including 18 organic acids, two sugars, one sugar alcohol, 20 amino acids, and five nucleobases, were identified through UHPLC-Q-Orbitrap-MS and HPLC data analyses (Tables S3–4). A heatmap was generated using features derived from the metabolite profile data, and hierarchical clustering was performed using the average component concentrations obtained from the metabolite profile results (Fig. 2A) to examine the changes in the overall metabolite composition of starter kimchi during 28 days of fermentation. The heat map shows the changes in the relative chemical composition of each metabolite type, emphasizing the differences associated with various fermentation stages and the application of different starters. Column clusters illustrate the distribution patterns of the metabolites. The sugar content decreased in all kimchi samples, whereas organic acid levels increased. Differences in the amino acid content were noted across all samples. The metabolic profile of kimchi was categorized into three distinct clusters, with LAB starters influencing fermentation throughout the study period. The 0-week samples were separated from the 2- and 4-week samples in the PCA, consistent with a previous report (Lee, Kim, Hwang, et al., 2024) (Fig. 2B). However, the 2- and 4-week samples exhibited more distinct variations based on the type of starter used rather than fermentation duration. Samples were primarily organized by fermentation stage in PC1, whereas they were distinctly grouped by the experimental group in PC2. Similar to the heatmap analysis results, three clusters emerged, with each cluster categorized into Groups I (0-week samples), II (a subset of 2- and 4-week samples: WiKim0176, WiKim39, WiKim0124, and the control), and III (another subset of 2- and 4-week samples: LP8, WiKim0189, WiKim0190, LC102, and LCM2). These were used to compare taste characteristics. This suggested that the choice of starter significantly influences the metabolic profile of kimchi throughout fermentation, highlighting the strain-specific metabolomic features of *Lactobacillaceae*. We conducted a combined analysis using an electronic tongue and nose to clarify which metabolites influenced the grouping.

#### 3.2.2 E-tongue analysis of starter kimchi flavor

E-tongue objectively assesses the taste by converting electrical signals into taste signals, offering high sensitivity and eliminating subjective sensory evaluation (Jiang et al., 2018). Flavor analysis of the starter kimchi was performed using a potentiometric chemical sensor, which is a widely used tool for e-tongues. This sensor detects the voltage difference between the working and reference electrodes, as the working electrode voltage fluctuates based on the analyte concentration (Ciosek & Wróblewski, 2011). The potentiometric E-tongue has been validated for its high accuracy compared to standard analytical methods and has been successfully applied to differentiate the flavor characteristics of various fermented foods, including commercial beers and wines (Tan & Xu, 2020). Fig. 2C shows the E-tongue response values for the sensory attributes of kimchi, including sourness (AHS), umami (NMS), sweetness (ANS), and bitterness (SCS), analyzed across Groups I, II, and III. Compared to Group I (week 0 kimchi), both Groups II and III showed changes in signal values during fermentation, including a significant increase in sourness, a decrease in bitterness, and statistically significant differences in umami between the two groups. Salinity (CTS) showed no significant difference. This suggested that the production of metabolites that contribute to the umami flavor of fermented kimchi varies depending on the starter strain, and required further investigation. Therefore, a correlation analysis was performed to determine the metabolites that contributed to the savory score.

#### 3.2.3 Correlation between metabolic profile and umami flavor perception in starter kimchi

**Fig. 3:**
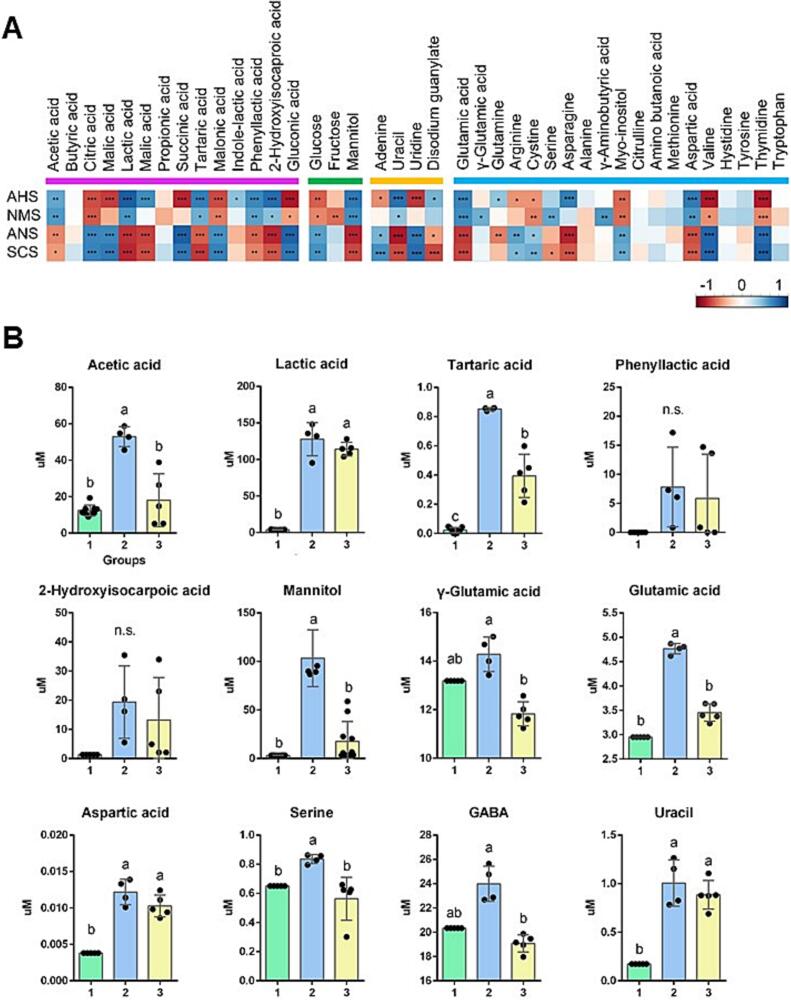
Integrated analysis of the metabolites and umami taste perception in kimchi via E-tongue evaluation. Four primary categories of physicochemical compounds—14 organic acids (magenta), three sugars (green), four nucleobases (yellow), and 19 amino acids (cyan)—were monitored throughout fermentation. The Spearman's rank correlation coefficient ranged from 1.0 to −1.0, corresponding to a strongly positive or negative correlation (*p < 0.01). SCS, bitterness; CTS, saltiness; NMS, umami; ANS, sweetness; AHS, sourness (A). Twelve key fermentative metabolites were identified via correlation analysis between E-tongue signal values and metabolite profiles (B). Different letters denote the significant differences (*p* < 0.05; ANOVA, followed by the Tukey HSD test). All experiments were conducted in triplicate. (For interpretation of the references to colour in this figure legend, the reader is referred to the web version of this article.)

Taste is a key factor that influences consumer acceptance and purchasing choices. In addition to sourness, sweetness, bitterness, and saltiness, umami is an essential taste quality. The relationship between the umami compound concentration and taste intensity measured using the E-tongue was analyzed in this study (Fig. 3A). The significantly correlated compounds were compared across Groups I, II, and III, as shown in Fig. 3B and Table S4. Twelve compounds had significantly positive correlations with the umami sensor signal values. These included five organic acids (acetic, lactic, tartaric, phenyllactic, and 2-hydroxyisocaproic acids), one sugar alcohol (mannitol), one nucleotide (uracil), and five amino acids (γ-glutamic acid, glutamic acid, aspartic acid, serine, and γ-aminobutyric acid [GABA]). These compounds generally increased throughout the kimchi fermentation process. However, acetic acid, tartaric acid, mannitol, γ-glutamic acid, glutamic acid, and GABA showed statistically significant differences between Groups II and III, with Group II showing higher concentrations than Group III. These findings explained the elevated umami perception observed in the E-tongue results of Group II. Organic acids are produced during sugar metabolism. A previous study demonstrated that LAB degrades glucose and fructose, yielding acidic end products, particularly lactic acid, which reduces pH (Park et al., 2018). Lactic acid is the most abundant compound produced during fermentation. This increase in sourness was primarily due to the significant production of acids. However, some organic acids, such as succinic and propionic acids found in Swiss cheese, mushrooms, and green tea, enhance the umami taste of food (Istiqamah et al., 2019). Fish sauce is commonly added to kimchi to enhance its umami flavor, and the acetic and lactic acids from salted fish play significant roles in taste enhancement (Park et al., 2001). This addition supports the breakdown of proteins in the fish, leading to the release of amino acids. Some free amino acids directly enhance taste, while others are precursors to bitter compounds (Lee et al., 2019). Naturally occurring umami compounds are found in various foods, including seafood, meat, vegetables, and fermented foods (Zhang et al., 2013). L-glutamic acid is recognized as a representative umami compound and highly sensitive oral sensory substance, exhibiting a low umami taste recognition threshold of 3.0 mmol kg^−1^ (Behrens et al., 2011). In addition, organic acids such as succinic and lactic acids contribute to the umami flavor and act synergistically with glutamic acid, intensifying its fresh and savory taste (Yang et al., 2024). Mannitol and l-serine, which both increase during kimchi fermentation (Eom et al., 2024), are considered key compounds that enhance sweet and umami flavors (Chen et al., 2023; Diepeveen et al., 2022). GABA is an essential inhibitory neurotransmitter in the mammalian brain known for its hypotensive, anti-inflammatory, and antidiabetic effects (Hou et al., 2024). GABA does not directly contribute to the umami taste but is derived from its precursor, L-glutamic acid, through an irreversible decarboxylation reaction catalyzed by glutamate decarboxylase (Pannerchelvan et al., 2023). The notable difference in GABA production between Groups II and III, which parallels the glutamate concentration, suggests active glutamate metabolism, as GABA is derived from its precursor L-glutamic acid through an irreversible decarboxylation reaction catalyzed by glutamate decarboxylase. Overall, these findings suggest that the distinct metabolite profile of Group II is associated with higher umami perception and more favorable sensory characteristics. Flavor enhancers are traditionally grouped into three primary types: amino acids and their sodium salts, nucleotides and their sodium salts, and organic acids and their sodium salts (Wang et al., 2021). In our dataset, nucleic acid-derived flavor enhancers increased throughout the fermentation period; however, no significant differences were observed between the groups.

#### 3.2.4 Analysis of aroma compounds in starter kimchi using an electronic nose

**Fig. 4:**
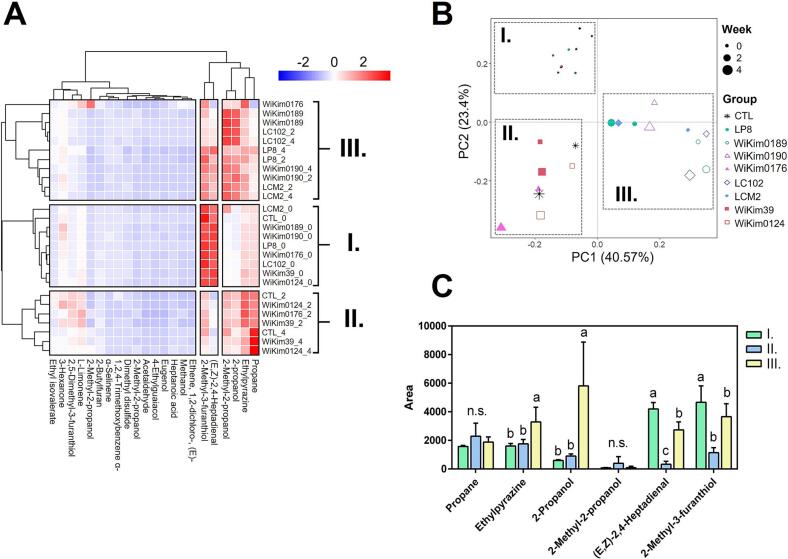
Aroma profile characterization of fermented kimchi using the E-nose technology. Hierarchical cluster analysis combined with a heatmap revealed the 22 key aroma compounds. The heatmap uses colour coding to represent relative abundance, with red indicating high abundance and blue indicating low abundance. Heat map (A). PCA plot (B). Six key fermentative aroma compounds were identified via correlation analysis of E-nose signal values (C). Different letters denote the significant differences (*p* < 0.05; ANOVA, followed by the Tukey HSD test). All experiments were conducted in triplicate. (For interpretation of the references to colour in this figure legend, the reader is referred to the web version of this article.)

Metal-oxide sensors, known for their high sensitivity and surface reactivity from free electrons and oxygen vacancies, are integral to electronic nose systems. These devices efficiently detect aroma patterns and volatile compounds, offering a practical alternative to complex chromatographic techniques for classifying diverse food products (Lu et al., 2022). Similar to the metabolite analysis, heat maps were generated to examine changes in the overall aroma profile composition of the starter kimchi during the 28-day fermentation. Hierarchical clustering and PCA were performed using the average component concentrations (Fig. 4A and B). The heat map and PCA revealed that clustering was driven more by differences in starter types than by fermentation stages, reflecting the three distinct clusters observed in the metabolite analysis and highlighting the aroma profile distribution patterns associated with each starter. Accordingly, these three clusters were classified into Groups I, II, and III to compare their aromatic characteristics. Six compounds were identified as key aroma components using clustering heatmap analysis. These compounds were propane, ethylpyrazine, 1,1-dichloroethene, 2-methyl-2-propanol, (E,Z)-2,4-heptadienal, and 2-methyl-3-furanthiol. Levels of propane, ethylpyrazine, and 1, 1-dichloroethene generally increase during kimchi fermentation. However, ethylpyrazine, 2-propanol, (E,Z)-2,4-heptadienal, and 2-methyl-3-furanthiol levels showed significant differences between Groups II and III. Pyrazines are key volatile compounds in fermented foods and are recognized for their characteristic roasted, nutty, earthy, and savory scents. They are vital in shaping the complex flavor profiles of fermented products such as soy sauce, fermented beans, cocoa, and certain cheeses, often enhancing umami perception and adding aromatic depth (Fayek et al., 2023). In this study, 2-propanol levels increased significantly after fermentation, especially in Group III. Although it does not directly impact food flavor, its similarity in structure and aroma to 1-propanol, which is known to mask ethereal and other odors, suggests that 2-propanol reduces off-flavors through a similar masking effect (Lee et al., 2021). A significant reduction in the levels of the fishy-smelling compound (E,Z)-2,4-heptadienal, originating from salted seafood (*jeotgal*) and a key ingredient in kimchi, was observed during fermentation, with the levels becoming nearly undetectable in Group II. This suggested that the microorganisms in Group II actively metabolize this compound or its precursors or generate metabolites that bind to and neutralize related substances, thereby inhibiting the development of fishy odors (Liu et al., 2025). Additionally, 2-methyl-3-furanthiol, a sulfur-containing compound, is known for its distinctive sulfurous and cabbage-like odor and can affect flavor even at very low concentrations (Yang et al., 2022). It has been consistently identified as a key contributor to the characteristic aroma of *Brassica* vegetables, which can be perceived as an off-flavor in kimchi (Wieczorek & Drabińska, 2022). The analysis of key volatile compounds revealed that starter strains significantly influence the aroma profile and sensory quality of kimchi during fermentation. Group II microorganisms exhibited a greater capacity to reduce off-flavor compounds, such as (E,Z)-2,4-heptadienal and 2-methyl-3-furanthiol, while enhancing desirable sensory attributes by modulating metabolites like ethylpyrazine. These findings suggest that selecting starter cultures with superior dominance and metabolic activity is critical for improving the flavor quality and consumer acceptability of fermented kimchi products.

## 4 Conclusion

This study investigated the effects of *Lactobacillaceae* starters on the flavor and aroma of kimchi, with a particular focus on flavor enhancement and sensory differentiation among strains. The inoculated starters produced unique metabolites compared to uninoculated kimchi. Notably, the effects on taste—including changes in amino acid profiles associated with umami perception and the rapid removal of off-flavors—varied among starter strains, highlighting the importance of careful starter selection. Overall, these findings provide valuable insights into dominant metabolites during fermentation under high-acid conditions and their impact on the sensory quality of kimchi. This study contributes to both scientific understanding and practical applications by elucidating the role of *Lactobacillaceae* starter strains in flavor preservation during long-term kimchi fermentation (*Mukeunji*).

## CRediT authorship contribution statement

**Moeun Lee:** Writing – review & editing, Writing – original draft, Visualization, Methodology, Investigation, Data curation, Conceptualization. **Daun Kim:** Methodology, Investigation, Data curation, Conceptualization. **Jung Hee Song:** Methodology, Investigation, Conceptualization. **In Min Hwang:** Methodology, Investigation, Formal analysis, Conceptualization. **So Yeong Mun:** Methodology, Investigation, Conceptualization. **Ji Yoon Chang:** Writing – review & editing, Supervision, Project administration, Funding acquisition, Conceptualization.

## Funding

This study was supported by the 10.13039/501100003722World Institute of Kimchi [grant number KE2501-1] and funded by the Ministry of Science and ICT, Republic of Korea.

## Declaration of competing interest

The authors declare that they have no known competing financial interests or personal relationships that could have appeared to influence the work reported in this paper.

## Data Availability

Data will be made available on request.
