# Supplementary material for: Comparative insights into the metabolite and taste discrepancies upon kimchi (Mukeunji) fermentation using different Lactobacillaceae starters
Source: Food Chem X. 2025 Sep 2;30:102982. doi: 10.1016/j.fochx.2025.102982 (PMC12451361; doi:10.1016/j.fochx.2025.102982)
Supplement: Supplementary material [file mmc1.docx]

Supplementary Table S1. Detailed analytical conditions of UHPLC-HESI-MS analysis.

| **Parameter** | **Details** |
| --- | --- |
| Instrument | Dionex Ultimate 3000 UHPLC + HESI Quadrupole-Orbitrap MS  (Thermo Scientific Inc., Bremen, Germany) |
| System control & data acquisition | Xcalibur^TM^4.5 software  (Thermo Fisher Scientific Inc., San Jose, CA, USA) |
| Quantitative data mode | Full MS scan mode |
| MS2 Resolution | 17,500 FWHM (m/z 200) |
| Column | Accucore aQ polar end-capped column (pre-column) + Hypersil GOLD C18 (primary column) |
| Mobile phase | Mobile phase A: 0.1% (v/v) formic acid in water  Mobile phase B: 0.1% (v/v) formic acid in acetonitrile |
| Gradient elution | 0–3.0 min: 0% B; 3.0–8.0 min: 45% B; 8.0–10.0 min: 100% B |
| Flow rate | 0.3 mL/min |
| Column oven temperature (°C) | 40 |
| Autosampler temperature (°C) | 10 |
| Injection volume | 2 μL |

Supplementary Table S2. Details of fermentative metabolite and mass spectrometry value.

| Compound name | Chemical formula | Molecular weight (g/mol) | Adduct | Precursor ions (m/z) | NCE^A^ | Fragments (m/z) ^B^ |
| --- | --- | --- | --- | --- | --- | --- |
| **Organic acid** |  |  |  |  |  |  |
| Gluconic acid | C_6_H_12_O_7_ | 196.2 | (M-H)- | 195.048 | 18 | 129.0165, 75.0063 |
| Indole-3-lactic acid | C_11_H_11_NO_3_ | 205.2 | (M-H)- | 204.0636 | 13 | 158.0582, 186.0529 |
| Leucic acid | C_6_H_12_O_3_ | 132.2 | (M-H)- | 131.0705 | 12 | 69.0365, 85.0671 |
| Phenyllactic acid | C_9_H_10_O_3_ | 166.2 | (M-H)- | 165.056 | 10 | 119.0502, 147.0452 |
| **Amino acid** |  |  |  |  |  |  |
| L-Alanine | C_3_H_7_NO_2_ | 89.1 | (M+H)+ | 90.05592 | 15 | 57.9359, 72.9057 |
| L-Arginine | C_6_H_14_N_4_O_2_ | 174.2 | (M+H)+ | 175.1195 | 18 | 70.0661, 130.0981 |
| L-Cystine | C_6_H_12_N_2_O_4_S_2_ | 240.3 | (M+H)+ | 241.0319 | 13 | 151.9840, 195.0266 |
| L-Glutamine | C_5_H_10_N_2_O_3_ | 146.1 | (M+H)+ | 147.0761 | 10 | 130.0497 |
| L-Serine | C_3_H_7_NO_3_ | 105.1 | (M+H)+ | 106.0507 | 10 | 60.0455, 88.0403 |
| L-Tyrosine | C_9_H_11_NO_3_ | 181.2 | (M+H)+ | 182.0819 | 13 | 136.0761, 147.0444, |
| L-Histidine | C_6_H_9_N_3_O_2_ | 155.2 | (M+H)+ | 156.0774 | 15 | 95.0613, 110.0721 |
| γ Glutamic acid | C_5_H_9_NO_4_ | 147.1 | (M+H)+ | 148.0609 | 10 | 102.0559, 130.0506 |
| y-Aminobutyric acid (GABA) | C_4_H_9_NO_2_ | 103.1 | (M+H)+ | 104.0714 | 18 | 87.0448, 69.0344 |
| Guanosine 5′-monophosphate disodium salt hydrate (GMP) | C_10_H_12_N_5_O_8_P•Na_2_ | 407.2 | (M-H)- | 362.053 | 10 | 78.959 |
| L-Asparagine | C4H8N2O3 | 132.1 | (M+H)+ | 133.0613 | 15 | 74.0246, 87.0563, 116.0350 |
| L-Citrulline | C6H13N3O3 | 175.2 | (M+H)+ | 176.1037 | 13 | 70.0661, 113.0716, 159.0769 |
| DL-2-Amino butanoic acid | C_4_H_9_NO_2_ | 103.1 | (M+H)+ | 104.0715 | 15 | 69.0346, 87.0450 |
| Methionine | C_5_H_11_NO_2_S | 149.2 | (M+H)+ | 150.0589 | 15 | 56.0506, 74.0610, 104.0536, 133.0323 |
| L -Aspartic acid | C_6_H_9_NO_5_ | 175.1 | (M-H)- | 174.041 | 10 | 88.0404 |
| Thymidine | C_10_H_14_N_2_O_5_ | 242.2 | (M-H)- | 241.0835 | 15 | 151.0513 |
| L-Tryptophan | C_11_H_12_N_2_O_2_ | 204.2 | (M+H)+ | 205.0976 | 13 | 146.0604, 159.0921, 188.0710 |
| ^A^NCE: normalized collision energy.  ^B^Fragments (m/z): The product ion with the highest intensity is underlined. | | | | | | |

Supplementary Table S3. Quantification of fermentative metabolites in kimchi during fermentation using HPLC.

| **Class** | **Compounds** | **CTL** | | | **LP8** | | | **WiKim0189** | | | |
| --- | --- | --- | --- | --- | --- | --- | --- | --- | --- | --- | --- |
|  |  | **0W** | **2W** | **4W** | **0W** | **2W** | **4W** | **0W** | **2W** | **4W** |  |
| Organic acids | Acetic acid | 13.37±0.08 | 57.22±0.15 | 52.30±0.14 | 13.27±0.17 | 34.16±0.20 | 43.48±0.4 | 13.20±0.08 | 23.34±0.13 | 29.37±0.30 |  |
|  | Butyric acid | 0.19±0.33 | 0.56±0.06 | 0.52±0.08 | 0.38±0.34 | 0.45±0.04 | N.D. | 0.57±0.07 | 0.44±0.08 | N.D. |  |
|  | Citric acid | 8.71±0.02 | 0.80±0.01 | 0.56±0.14 | 8.85±0.09 | 0.80±0.01 | 0.69±0.00 | 8.74±0.12 | 0.84±0.01 | 0.71±0.00 |  |
|  | D-Malic acid | 95.39±0.29 | 77.63±0.41 | 66.41±0.34 | 89.92±0.81 | 24.26±0.04 | 4.18±0.08 | 90.21±0.74 | 6.70±0.12 | 4.21±0.08 |  |
|  | Lactic acid | 4.37±0.04 | 97.06±0.54 | 93.57±2.65 | 4.17±0.06 | 81.04±0.11 | 134.99±0.26 | 4.08±0.11 | 103.83±0.22 | 152.33±0.84 |  |
|  | L-Malic acid | N.D. | 3.42±0.10 | 3.54±0.08 | N.D. | 2.68±0.25 | 2.05±0.03 | N.D. | 3.09±0.27 | 3.38±0.13 |  |
|  | Propionic acid | 72.54±0.20 | 70.67±0.51 | 64.65±0.49 | 74.48±0.67 | 72.48±0.11 | 1.46±0.13 | 73.98±0.88 | 76.36±0.20 | 2.01±0.22 |  |
|  | Succinic acid | 26.26±0.11 | 5.21±0.09 | 3.37±0.06 | 25.49±0.30 | 2.91±0.05 | 3.79±0.13 | 25.18±0.47 | 2.84±0.10 | 4.32±0.09 |  |
|  | Tartaric acid | 0.05±0.01 | 0.74±0.01 | 0.98±0.02 | 0.04±0.04 | 0.32±0.28 | 3.94±0.02 | 0.04±0.00 | 0.56±0.04 | 3.32±0.02 |  |
|  | Malonic acid | 14.77±0.03 | 10.03±0.15 | 8.54±0.12 | 14.90±0.11 | 7.71±0.15 | 4.18±0.02 | 14.73±0.19 | 5.60±0.10 | 6.64±0.05 |  |
| Sugar | Glucose | 97.74±0.88 | 63.24±0.17 | 12.50±0.41 | 97.99±0.89 | 75.65±0.02 | 53.68±0.03 | 97.23±0.02 | 60.98±0.03 | 20.45±0.03 |  |
|  | Fructose | 111.74±0.01 | 32.37±0.02 | 23.32±0.03 | 111.74±0.02 | 75.68±0.01 | 55.45±0.02 | 117.72±0.03 | 102.35±0.01 | 103.98±0.03 |  |
|  | Mannitol | 3.25±0.02 | 89.86±0.03 | 89.16±0.01 | 3.24±0.02 | 48.60±0.01 | 58.86±0.01 | 3.22±0.03 | 20.40±0.03 | 22.71±0.02 |  |

Supplementary Table S3. Continued

| **Class** | **Compounds** | **WiKim0190** | | | **WiKim0176** | | |
| --- | --- | --- | --- | --- | --- | --- | --- |
|  |  | **0W** | **2W** | **4W** | **0W** | **2W** | **4W** |
| Organic acids | Acetic acid | 10.45±0.09 | 5.57±0.09 | 4.81±0.87 | 10.80±0.10 | 18.40±0.17 | 23.09±0.26 |
|  | Butyric acid | 0.34±0.30 | 0.27±0.03 | 0.16±0.01 | 0.52±0.07 | 0.44±0.04 | 0.23±0.01 |
|  | Citric acid | 8.28±0.01 | 7.13±0.12 | 3.48±4.73 | 8.14±0.65 | 0.65±0.00 | 0.69±0.00 |
|  | D-Malic acid | 87.22±0.14 | 59.28±1.10 | 4.19±0.36 | 85.97±0.08 | 69.69±0.22 | 5.71±0.06 |
|  | Lactic acid | 3.76±0.07 | 69.78±1.13 | 137.62±0.25 | 3.75±0.08 | 94.59±0.28 | 176.09±0.17 |
|  | L-Malic acid | N.D. | 2.39±0.06 | 3.93±0.14 | N.D. | 3.21±0.03 | N.D. |
|  | Propionic acid | 68.62±0.09 | 72.38±1.44 | 3.22±0.07 | 67.70±0.01 | 65.29±0.25 | 0.99±0.11 |
|  | Succinic acid | 24.51±0.03 | 8.12±0.19 | 5.65±0.43 | 24.78±0.03 | 3.54±0.02 | 12.33±0.02 |
|  | Tartaric acid | 0.03±0.00 | N.D. | 0.59±0.01 | 0.02±0.00 | 0.70±0.00 | 0.97±0.00 |
|  | Malonic acid | 14.33±0.10 | 9.17±0.19 | 7.94±0.02 | 14.04±0.07 | 8.66±0.05 | 1.45±0.01 |
| Sugar | Glucose | 97.23±0.03 | 87.39±0.03 | 93.96±0.02 | 97.24±0.01 | 59.04±0.02 | -0.03±0.03 |
|  | Fructose | 111.72±0.03 | 112.64±0.02 | 118.75±0.01 | 111.72±0.03 | 93.55±0.01 | -0.01±0.01 |
|  | Mannitol | 3.25±0.02 | 3.27±0.02 | 3.51±0.03 | 3.23±0.03 | 10.28±0.02 | 16.64±0.04 |

Supplementary Table S3. Continued

| **Class** | **Compounds** | **LC102** | | | **LCM2** | | |
| --- | --- | --- | --- | --- | --- | --- | --- |
|  |  | **0W** | **2W** | **4W** | **0W** | **2W** | **4W** |
| Organic acids | Acetic acid | 11.28±0.12 | 13.88±0.06 | 15.69±0.19 | 19.31±0.20 | 5.12±0.04 | 5.29±0.76 |
|  | Butyric acid | 0.31±0.27 | 0.31±0.04 | 0.18±0.00 | 0.53±0.06 | 0.27±0.03 | 0.21±0.01 |
|  | Citric acid | 7.67±0.02 | 0.80±0.01 | 0.72±0.00 | 7.88±0.04 | 6.67±0.03 | 4.03±5.43 |
|  | D-Malic acid | 80.34±0.21 | 52.89±0.29 | 4.25±0.06 | 85.97±0.08 | 54.16±0.19 | 4.67±0.29 |
|  | Lactic acid | 3.70±0.10 | 90.96±0.36 | 140.56±0.17 | 3.75±0.08 | 71.65±0.18 | 157.65±0.09 |
|  | L-Malic acid | N.D. | 2.82±0.02 | 3.51±0.02 | N.D. | 2.58±0.00 | 4.75±0.15 |
|  | Propionic acid | 64.26±0.11 | 76.32±0.38 | 2.72±0.25 | 65.39±0.26 | 72.88±0.24 | 3.10±0.00 |
|  | Succinic acid | 22.04±0.08 | 2.84±0.04 | 3.38±0.05 | 22.04±0.08 | 2.98±0.03 | 3.91±0.41 |
|  | Tartaric acid | 0.02±0.00 | 0.45±0.00 | 0.75±0.00 | N.D. | N.D. | 0.90±0.00 |
|  | Malonic acid | 13.49±0.03 | 8.60±0.04 | 6.95±0.01 | 10.93±0.09 | 9.13±0.02 | 9.61±0.00 |
| Sugar | Glucose | 97.25±0.00 | 85.00±0.02 | 78.32±0.04 | 97.25±0.00 | 90.96±0.01 | 93.90±0.04 |
|  | Fructose | 111.73±0.02 | 113.18±0.00 | 110.01±0.03 | 111.73±0.02 | 124.37±0.02 | 127.23±0.02 |
|  | Mannitol | 3.25±0.02 | 5.68±0.03 | 6.71±0.02 | 3.25±0.01 | 3.31±0.03 | 3.34±0.03 |

Supplementary Table S3. Continued

| **Class** | **Compounds** | **WiKim39** | | | **WiKim0124** | | | |
| --- | --- | --- | --- | --- | --- | --- | --- | --- |
|  |  | **0W** | **2W** | **4W** | **0W** | **2W** | **4W** |  |
| Organic acids | Acetic acid | 9.00±0.11 | 21.09±0.12 | 70.10±0.57 | 10.98±0.10 | 53.63±0.10 | 63.71±0.09 |  |
|  | Butyric acid | 0.13±0.23 | 0.63±0.09 | N.D. | 0.13±0.22 | 0.44±0.05 | N.D. |  |
|  | Citric acid | 7.45±0.02 | 3.73±0.04 | 0.67±0.01 | 7.24±0.03 | 0.58±0.11 | 0.63±0.00 |  |
|  | D-Malic acid | 80.34±0.08 | 2.91±0.15 | 5.78±0.11 | 77.70±0.24 | 65.84±0.16 | 5.58±0.03 |  |
|  | Lactic acid | 3.75±0.08 | 117.18±0.22 | 179.01±0.40 | 3.54±0.10 | 83.76±0.09 | 180.09±0.43 |  |
|  | L-Malic acid | N.D. | 3.29±0.25 | 1.15±0.01 | N.D. | 3.03±0.09 | 0.63±0.55 |  |
|  | Propionic acid | 62.97±0.08 | 59.15±0.21 | N.D. | 61.29±0.12 | 62.54±0.29 | N.D. |  |
|  | Succinic acid | 22.04±0.08 | 6.40±0.05 | 5.47±0.07 | 21.24±0.05 | 3.86±0.07 | 6.11±0.23 |  |
|  | Tartaric acid | N.D. | 0.552±0.00 | 1.15±0.01 | N.D. | 0.63±0.01 | 1.08±0.00 |  |
|  | Malonic acid | 13.36±0.05 | 7.27±0.15 | 2.32±0.02 | 12.82±0.05 | 8.60±0.09 | 1.97±0.01 |  |
| Sugar | Glucose | 97.23±0.03 | 84.07±0.02 | 31.49±0.04 | 97.24±0.02 | 65.58±0.03 | 7.30±0.02 |  |
|  | Fructose | 111.73±0.02 | 109.33±0.01 | 33.68±0.03 | 111.74±0.01 | 31.51±0.02 | 15.75±0.02 |  |
|  | Mannitol | 3.26±0.01 | 44.12±0.03 | 155.31±0.01 | 3.25±0.01 | 95.38±0.02 | 86.72±0.02 |  |

^A^N.D.: Not detected

Supplementary Table S4. Quantitative identification of fermentative metabolites in kimchi during fermentation using LC-Q-Orbitrap MS.

| **Class** | **Tentative identification** | **CTL (mM)** | | | **LP8 (mM)** | | | **WiKim0189 (mM)** | | |
| --- | --- | --- | --- | --- | --- | --- | --- | --- | --- | --- |
|  |  | **0W** | **2W** | **4W** | **0W** | **2W** | **4W** | **0W** | **2W** | **4W** |
| Organic acids | 2-Hydroxyisocaproic acid | 1.37±0.42 | 20.19±4.54 | 20.60±5.41 | 1.37±0.42 | 29.36±6.69 | 38.68±7.65 | 1.37±0.42 | 22.16±7.60 | 23.97±4.98 |
|  | Indole-3-lactic acid | 0.02±0.00 | 0.03±0.02 | 0.04±0.03 | 0.02±0.00 | 0.11±0.02 | 0.15±0.02 | 0.02±0.00 | 0.18±0.06 | 0.22±0.04 |
|  | Phenyllactic acid | 0.00±0.00 | 7.00±2.14 | 7.55±2.53 | 0.00±0.00 | 11.76±1.72 | 15.52±1.73 | 0.00±0.00 | 14.75±3.07 | 14.65±3.70 |
|  | Gluconic acid | 1.41±0.34 | 0.31±0.04 | 0.28±0.07 | 1.41±0.34 | 0.23±0.04 | 0.23±0.04 | 1.41±0.34 | 0.22±0.03 | 0.23±0.04 |
| Amino acid | L-Alanine | 1.18±0.10 | 1.19±0.11 | 1.24±0.10 | 1.18±0.10 | 1.22±0.09 | 1.25±0.14 | 1.18±0.10 | 1.25±0.19 | 1.20±0.06 |
|  | L-Arginine | 0.96±0.09 | 0.15±0.02 | 0.08±0.01 | 0.96±0.09 | 0.10±0.02 | 0.08±0.01 | 0.96±0.09 | 0.67±0.14 | 0.69±0.10 |
|  | L-Cystine | 5.45±1.06 | 4.44±0.76 | 4.42±0.60 | 5.45±1.06 | 4.27±0.77 | 4.42±0.96 | 5.45±1.06 | 4.53±1.33 | 4.33±0.71 |
|  | L-Glutamine | 0.73±0.11 | 0.82±0.11 | 0.81±0.13 | 0.73±0.11 | 0.72±0.10 | 0.77±0.12 | 0.73±0.11 | 0.74±0.16 | 0.78±0.10 |
|  | L-Serine | 0.65±0.04 | 0.85±0.08 | 0.86±0.07 | 0.65±0.04 | 0.45±0.03 | 0.15±0.01 | 0.65±0.04 | 0.78±0.05 | 0.73±0.07 |
|  | L-Tyrosine | 7.76±1.59 | 8.24±1.54 | 6.56±1.47 | 7.76±1.59 | 7.66±1.51 | 8.71±1.66 | 7.76±1.59 | 6.93±2.18 | 8.18±1.49 |
|  | L-Histidine | 7.45±0.45 | 7.14±0.81 | 6.05±1.46 | 7.45±0.45 | 7.05±1.37 | 8.09±1.16 | 7.45±0.45 | 7.22±0.64 | 7.44±1.41 |
|  | Glutamic acid | 2.95±0.37 | 4.69±0.66 | 5.04±0.58 | 2.95±0.37 | 3.19±0.33 | 3.27±0.51 | 2.95±0.37 | 3.46±0.42 | 3.30±0.48 |
|  | Gamma glutamic acid | 13.19±0.68 | 13.75±1.66 | 13.09±2.50 | 13.19±0.68 | 11.45±0.71 | 11.02±0.70 | 13.19±0.68 | 11.63±0.43 | 11.57±0.42 |
|  | GABA | 20.33±4.45 | 23.74±3.52 | 21.98±4.16 | 20.33±4.45 | 17.49±2.02 | 18.44±1.16 | 20.33±4.45 | 18.55±2.06 | 19.35±0.92 |
|  | Asparagine | 2.86±0.25 | 4.46±0.28 | 4.27±0.48 | 2.86±0.25 | 3.29±0.18 | 3.37±0.23 | 2.86±0.25 | 3.12±0.21 | 3.19±0.45 |
|  | Citrulline | 0.92±0.12 | 0.66±0.10 | 0.59±0.10 | 0.92±0.12 | 0.46±0.12 | 0.33±0.21 | 0.92±0.12 | 0.88±0.18 | 0.89±0.13 |
|  | Amino butanoic acid | 8.68±0.55 | 9.51±0.50 | 9.26±0.51 | 8.68±0.55 | 7.68±0.44 | 7.68±0.51 | 8.68±0.55 | 7.44±1.29 | 7.76±0.53 |
|  | Methionine | 0.40±0.06 | 0.49±0.07 | 0.48±0.09 | 0.40±0.06 | 0.41±0.06 | 0.46±0.05 | 0.40±0.06 | 0.36±0.10 | 0.41±0.06 |
|  | Aspartic acid | 0.00±0.00 | 0.01±0.00 | 0.01±0.00 | 0.00±0.00 | 0.01±0.00 | 0.01±0.00 | 0.00±0.00 | 0.01±0.00 | 0.01±0.00 |
|  | Thymidine | 0.09±0.02 | 0.00±0.00 | 0.00±0.00 | 0.09±0.02 | 0.00±0.00 | 0.00±0.00 | 0.09±0.02 | 0.00±0.00 | 0.00±0.00 |
|  | Tryptophan | 0.39±0.06 | 0.40±0.07 | 0.16±0.04 | 0.39±0.06 | 0.49±0.09 | 0.51±0.08 | 0.39±0.06 | 0.45±0.14 | 0.45±0.09 |
| Nucleobase | Adenine | 0.20±0.06 | 0.12±0.03 | 0.19±0.03 | 0.20±0.06 | 0.05±0.02 | 0.11±0.04 | 0.20±0.06 | 0.05±0.02 | 0.07±0.05 |
|  | Guanosine monophosphate | 1.50±0.31 | 1.59±0.34 | 1.64±0.33 | 1.50±0.31 | 1.49±0.33 | 1.41±0.33 | 1.50±0.31 | 1.62±0.31 | 1.55±0.32 |
|  | Uracil | 0.17±0.04 | 1.27±0.20 | 1.26±0.24 | 0.17±0.04 | 1.06±0.19 | 1.15±0.20 | 0.17±0.04 | 0.85±0.26 | 0.91±0.15 |
|  | Uridine | 0.44±0.11 | 0.01±0.00 | 0.04±0.01 | 0.44±0.11 | 0.00±0.00 | 0.01±0.00 | 0.44±0.11 | 0.01±0.00 | 0.00±0.00 |
| ^A^N.D.: Not detected | | | | | | | | | | |

Supplementary Table S4. Continued.

| **Class** | **Tentative identification** | **WiKim0190 (mM)** | | | **WiKim0176 (mM)** | | |
| --- | --- | --- | --- | --- | --- | --- | --- |
|  |  | **0W** | **2W** | **4W** | **0W** | **2W** | **4W** |
| Organic acids | 2-Hydroxyisocaproic acid | 1.37±0.42 | 2.11±0.32 | 2.12±0.45 | 1.37±0.42 | 4.54±1.04 | 6.54±1.76 |
|  | Indole-3-lactic acid | 0.02±0.00 | 0.02±0.00 | 0.02±0.00 | 0.02±0.00 | 0.02±0.00 | 0.03±0.01 |
|  | Phenyllactic acid | 0.00±0.00 | 0.00±0.00 | 0.00±0.00 | 0.00±0.00 | 0.37±0.41 | 1.23±0.75 |
|  | Gluconic acid | 1.41±0.34 | 0.95±0.14 | 1.00±0.10 | 1.41±0.34 | 0.82±0.05 | 0.75±0.06 |
| Amino acid | L-Alanine | 1.18±0.10 | 1.25±0.10 | 1.30±0.14 | 1.18±0.10 | 1.15±0.10 | 1.16±0.13 |
|  | L-Arginine | 0.96±0.09 | 0.70±0.08 | 0.69±0.09 | 0.96±0.09 | 0.78±0.11 | 0.75±0.11 |
|  | L-Cystine | 5.45±1.06 | 5.22±0.69 | 5.12±1.00 | 5.45±1.06 | 4.47±0.61 | 4.11±0.32 |
|  | L-Glutamine | 0.73±0.11 | 0.71±0.07 | 0.73±0.10 | 0.73±0.11 | 0.72±0.11 | 0.72±0.13 |
|  | L-Serine | 0.65±0.04 | 0.59±0.06 | 0.63±0.07 | 0.65±0.04 | 0.85±0.07 | 0.74±0.04 |
|  | L-Tyrosine | 7.76±1.59 | 8.50±0.97 | 8.66±0.73 | 7.76±1.59 | 9.25±0.86 | 11.59±2.02 |
|  | L-Histidine | 7.45±0.45 | 7.80±0.90 | 8.12±1.12 | 7.45±0.45 | 8.47±0.71 | 10.56±1.95 |
|  | Glutamic acid | 2.95±0.37 | 3.32±0.46 | 3.48±0.46 | 2.95±0.37 | 4.65±0.54 | 4.58±0.56 |
|  | Gamma glutamic acid | 13.19±0.68 | 11.42±0.26 | 12.03±0.41 | 13.19±0.68 | 13.86±0.24 | 14.13±0.84 |
|  | GABA | 20.33±4.45 | 18.91±0.64 | 20.01±0.54 | 20.33±4.45 | 23.45±0.30 | 21.90±4.23 |
|  | GMP | 1.50±0.31 | 1.55±0.27 | 1.66±0.36 | 1.50±0.31 | 1.86±0.41 | 2.28±0.59 |
|  | Asparagine | 2.86±0.25 | 3.08±0.40 | 2.96±0.17 | 2.86±0.25 | 3.20±0.20 | 3.06±0.48 |
|  | Citrulline | 0.92±0.12 | 0.95±0.12 | 0.94±0.11 | 0.92±0.12 | 1.15±0.16 | 1.00±0.15 |
|  | Amino butanoic acid | 8.68±0.55 | 7.99±0.29 | 8.02±0.41 | 8.68±0.55 | 8.48±0.65 | 8.49±0.73 |
|  | Methionine | 0.40±0.06 | 0.40±0.02 | 0.42±0.05 | 0.40±0.06 | 0.42±0.07 | 0.43±0.07 |
|  | Aspartic acid | 0.00±0.00 | 0.01±0.00 | 0.01±0.00 | 0.00±0.00 | 0.01±0.00 | 0.01±0.00 |
|  | Thymidine | 0.09±0.02 | 0.00±0.00 | 0.00±0.00 | 0.09±0.02 | 0.00±0.00 | 0.00±0.00 |
|  | Tryptophan | 0.39±0.06 | 0.24±0.03 | 0.20±0.03 | 0.39±0.06 | 0.34±0.06 | 0.27±0.06 |
| Nucleobase | Adenine | 0.20±0.06 | 0.06±0.03 | 0.05±0.02 | 0.20±0.06 | 0.08±0.01 | 0.15±0.06 |
|  | Uracil | 0.17±0.04 | 0.68±0.07 | 0.69±0.10 | 0.17±0.04 | 0.80±0.15 | 0.76±0.16 |
|  | Uridine | 0.44±0.11 | 0.03±0.00 | 0.03±0.02 | 0.44±0.11 | 0.04±0.00 | 0.05±0.02 |
| ^A^N.D.: Not detected | | | | | | | |

Supplementary Table S4. Continued.

| **Class** | **Tentative identification** | **LC102 (mM)** | | | **LCM2 (mM)** | | |
| --- | --- | --- | --- | --- | --- | --- | --- |
|  |  | **0W** | **2W** | **4W** | **0W** | **2W** | **4W** |
| Organic acids | 2-Hydroxyisocaproic acid | 1.37±0.42 | 4.68±0.97 | 5.16±1.75 | 1.37±0.42 | 1.95±0.40 | 2.22±0.73 |
|  | Indole-3-lactic acid | 0.02±0.00 | 0.03±0.01 | 0.03±0.01 | 0.02±0.00 | 0.02±0.00 | - |
|  | Phenyllactic acid | 0.00±0.00 | 0.74±0.41 | 1.00±0.80 | 0.00±0.00 | 0.00±0.00 | 0.00±0.00 |
|  | Gluconic acid | 1.41±0.34 | 0.55±0.05 | 0.56±0.08 | 1.41±0.34 | 0.85±0.11 | 0.90±0.05 |
| Amino acid | L-Alanine | 1.18±0.10 | 1.32±0.06 | 1.41±0.16 | 1.18±0.10 | 1.29±0.09 | 1.31±0.13 |
|  | L-Arginine | 0.96±0.09 | 0.83±0.06 | 0.87±0.07 | 0.96±0.09 | 0.72±0.08 | 0.73±0.12 |
|  | L-Cystine | 5.45±1.06 | 5.36±1.24 | 4.73±0.97 | 5.45±1.06 | 5.37±0.72 | 5.31±1.15 |
|  | L-Glutamine | 0.73±0.11 | 0.80±0.08 | 0.81±0.17 | 0.73±0.11 | 0.78±0.10 | 0.80±0.15 |
|  | L-Serine | 0.65±0.04 | 0.81±0.09 | 0.84±0.05 | 0.65±0.04 | 0.63±0.08 | 0.61±0.03 |
|  | L-Tyrosine | 7.76±1.59 | 0.38±0.10 | 0.70±0.26 | 7.76±1.59 | 8.31±1.10 | 10.47±1.53 |
|  | L-Histidine | 7.45±0.45 | 0.39±0.32 | 0.87±0.29 | 7.45±0.45 | 7.76±0.94 | 9.36±1.65 |
|  | Glutamic acid | 2.95±0.37 | 3.64±0.47 | 3.62±0.42 | 2.95±0.37 | 3.65±0.51 | 3.63±0.39 |
|  | Gamma-glutamic acid | 13.19±0.68 | 12.18±1.05 | 12.96±0.96 | 13.19±0.68 | 11.92±0.66 | 12.10±0.55 |
|  | GABA | 20.33±4.45 | 19.28±3.96 | 20.44±3.38 | 20.33±4.45 | 20.04±1.11 | 18.27±3.78 |
|  | GMP | 1.50±0.31 | 1.67±0.34 | 1.72±0.45 | 1.50±0.31 | 1.76±0.44 | 1.72±0.36 |
|  | Asparagine | 2.86±0.25 | 3.23±0.46 | 3.17±0.19 | 2.86±0.25 | 3.27±0.38 | 3.11±0.28 |
|  | Citrulline | 0.92±0.12 | 0.98±0.13 | 1.01±0.18 | 0.92±0.12 | 0.98±0.11 | 1.00±0.18 |
|  | Amino butanoic acid | 8.68±0.55 | 8.06±0.77 | 8.14±0.98 | 8.68±0.55 | 7.79±0.71 | 8.07±0.84 |
|  | Methionine | 0.40±0.06 | 0.41±0.05 | 0.43±0.10 | 0.40±0.06 | 0.41±0.06 | 0.46±0.10 |
|  | Acetyl aspartic acid | 0.00±0.00 | 0.01±0.00 | 0.01±0.00 | 0.00±0.00 | 0.01±0.00 | 0.01±0.00 |
|  | Thymidine | 0.09±0.02 | 0.00±0.00 | 0.00±0.00 | 0.09±0.02 | 0.00±0.00 | 0.00±0.00 |
|  | Tryptophan | 0.39±0.06 | 0.26±0.05 | 0.20±0.06 | 0.39±0.06 | 0.25±0.04 | 0.22±0.06 |
| Nucleobase | Adenine | 0.20±0.06 | 0.07±0.03 | 0.06±0.02 | 0.20±0.06 | 0.05±0.03 | 0.05±0.03 |
|  | Uracil | 0.17±0.04 | 0.86±0.13 | 0.88±0.25 | 0.17±0.04 | 0.85±0.11 | 0.92±0.22 |
|  | Uridine | 0.44±0.11 | 0.01±0.00 | 0.01±0.00 | 0.44±0.11 | 0.01±0.00 | 0.00±0.00 |
| ^A^N.D.: Not detected | | | | | | | |

Supplementary Table S4. Continued.

| **Class** | **Tentative identification** | **WK39 (mM)** | | | **WK0124 (mM)** | | |
| --- | --- | --- | --- | --- | --- | --- | --- |
|  |  | **0W** | **2W** | **4W** | **0W** | **2W** | **4W** |
| Organic acids | 2-Hydroxyisocaproic acid | 1.37±0.42 | 36.64±7.02 | 34.30±8.49 | 1.37±0.42 | 19.07±3.98 | 13.29±5.01 |
|  | Indole-3-lactic acid | 0.02±0.00 | 0.17±0.17 | 0.27±0.05 | 0.02±0.00 | 0.03±0.01 | 0.03±0.01 |
|  | Phenyllactic acid | 0.00±0.00 | 18.11±4.05 | 16.25±3.70 | 0.00±0.00 | 6.61±1.86 | 5.55±1.12 |
|  | Gluconic acid | 1.41±0.34 | 0.51±0.10 | 0.57±0.12 | 1.41±0.34 | 0.29±0.07 | 0.23±0.05 |
| Amino acid | L-Alanine | 1.18±0.10 | 1.18±0.12 | 1.20±0.14 | 1.18±0.10 | 1.21±0.11 | 1.17±0.05 |
|  | L-Arginine | 0.96±0.09 | 1.09±0.14 | 0.96±0.11 | 0.96±0.09 | 0.05±0.01 | 0.04±0.01 |
|  | L-Cystine | 5.45±1.06 | 4.33±0.58 | 4.16±0.79 | 5.45±1.06 | 4.41±0.47 | 3.47±1.01 |
|  | L-Glutamine | 0.73±0.11 | 0.78±0.09 | 0.76±0.11 | 0.73±0.11 | 0.83±0.13 | 0.69±0.14 |
|  | L-Serine | 0.65±0.04 | 0.82±0.09 | 0.84±0.08 | 0.65±0.04 | 0.84±0.06 | 0.88±0.11 |
|  | L-Tyrosine | 7.76±1.59 | 10.25±0.92 | 10.05±1.19 | 7.76±1.59 | 9.84±1.44 | 6.94±0.88 |
|  | L-Histidine | 7.45±0.45 | 9.25±0.74 | 8.52±0.91 | 7.45±0.45 | 7.85±0.53 | 6.55±0.51 |
|  | Glutamic acid | 2.95±0.37 | 4.65±0.56 | 4.96±0.59 | 2.95±0.37 | 4.68±0.62 | 4.87±0.42 |
|  | Gamma glutamic acid | 13.19±0.68 | 14.74±0.96 | 15.28±0.65 | 13.19±0.68 | 15.00±0.85 | 14.36±0.56 |
|  | GABA | 20.33±4.45 | 25.17±1.63 | 26.10±1.18 | 20.33±4.45 | 25.10±1.02 | 24.42±1.04 |
|  | GMP | 1.50±0.31 | 1.59±0.37 | 1.66±0.33 | 1.50±0.31 | 1.58±0.33 | 1.69±0.39 |
|  | Asparagine | 2.86±0.25 | 3.04±0.33 | 3.12±0.32 | 2.86±0.25 | 2.60±0.28 | 4.87±0.61 |
|  | Citrulline | 0.92±0.12 | 1.19±0.15 | 1.03±0.17 | 0.92±0.12 | 0.65±0.11 | 0.49±0.06 |
|  | Amino butanoic acid | 8.68±0.55 | 10.02±0.63 | 9.52±0.84 | 8.68±0.55 | 9.68±0.69 | 7.50±2.14 |
|  | Methionine | 0.40±0.06 | 0.47±0.04 | 0.45±0.08 | 0.40±0.06 | 0.53±0.08 | 0.36±0.14 |
|  | Acetyl aspartic acid | 0.00±0.00 | 0.01±0.00 | 0.01±0.00 | 0.00±0.00 | 0.01±0.00 | 0.01±0.01 |
|  | Thymidine | 0.09±0.02 | 0.00±0.00 | 0.00±0.00 | 0.09±0.02 | 0.00±0.00 | 0.00±0.00 |
|  | Tryptophan | 0.39±0.06 | 0.30±0.05 | 0.07±0.02 | 0.39±0.06 | 0.50±0.09 | 0.19±0.03 |
| Nucleobase | Adenine | 0.20±0.06 | 0.06±0.01 | 0.11±0.03 | 0.20±0.06 | 0.12±0.07 | 0.08±0.01 |
|  | Uracil | 0.17±0.04 | 0.72±0.11 | 0.94±0.22 | 0.17±0.04 | 1.32±0.20 | 0.98±0.11 |
|  | Uridine | 0.44±0.11 | 0.15±0.03 | 0.05±0.01 | 0.44±0.11 | 0.01±0.00 | 0.01±0.01 |
| ^A^N.D.: Not detected | | | | | | | |
